# Botanical Text-Book

**Published:** 1858-03

**Authors:** 


ART. VIII.—*Introduction to Structural and Systematic Botany, and Vegetable Physiology: being a Fifth and Revised Edition of the Botanical Text-Book.* By ASA GRAY, M.D., Fisher Professor of Natural History in Harvard University. 8vo. pp. 555. Ivison and Phinney: New York, 1858.

As it does not come within our province to review at length, oftener than occasionally, works upon subjects that are only collateral to medicine, and having recently furnished our readers with such a review of some of Dr. Gray's contributions to botanical literature, we are reluctantly compelled to give the above book only a passing notice. Of the general character and scope of the work all who are interested in such matters are sufficiently informed, the present being the fifth edition of what was formerly entitled the Botanical Text-Book; and we would, therefore, simply say, that it has undergone such a revision as the advancing state of the science demands, and is undoubtedly the best work on the subject in the English or, according to Professor Agassiz, in any other language.

Of the mechanical execution of the book—the paper, printing, and illustrations, over thirteen hundred in number—we can speak in the same unqualified terms. It is one of the most finished specimens of the typographical art that we have ever examined, and the mere handling of the book and running the eye over its beautiful pages are productive of a species of pleasure that we have seldom before experienced.

